# Comparative genomics of rainbow trout (*Oncorhynchus mykiss*): Is the genetic architecture of migratory behavior conserved among populations?

**DOI:** 10.1002/ece3.10241

**Published:** 2023-06-26

**Authors:** Catherine I. Clare, Krista M. Nichols, Frank P. Thrower, Ewann A. Berntson, Matthew C. Hale

**Affiliations:** ^1^ Department of Biology Texas Christian University Fort Worth Texas USA; ^2^ Conservation Biology Division, Northwest Fisheries Science Center National Marine Fisheries Service, National Oceanic and Atmospheric Administration Seattle Washington USA; ^3^ Ted Stevens Marine Research Institute, Alaska Fisheries Science Center, NOAA Juneau Alaska USA

**Keywords:** genomics, migration, pooled sequencing, rainbow trout, steelhead trout

## Abstract

Rainbow trout (*Oncorhynchus mykiss*) are a partially migratory species wherein some individuals undergo long‐distance anadromous migrations, and others stay as residents in their native freshwater streams. The decision to migrate is known to be highly heritable, and yet, the underlying genes and alleles associated with migration are not fully characterized. Here we used a pooled approach of whole‐genome sequence data from migratory and resident trout of two native populations—Sashin Creek, Alaska and Little Sheep Creek, Oregon—to obtain a genome‐wide perspective of the genetic architecture of resident and migratory life history. We calculated estimates of genetic differentiation, genetic diversity, and selection between the two phenotypes to locate regions of interest and then compared these associations between populations. We identified numerous genes and alleles associated with life history development in the Sashin Creek population with a notable area on chromosome 8 that may play a critical role in the development of the migratory phenotype. However, very few alleles appeared to be associated with life history development in the Little Sheep Creek system, suggesting population‐specific genetic effects are likely important in the development of anadromy. Our results indicate that a migratory life history is not controlled by a singular gene or region but supports the idea that there are many independent ways for a migratory phenotype to emerge in a population. Therefore, conserving and promoting genetic diversity in migratory individuals is paramount to conserving these populations. Ultimately, our data add to a growing body of literature that suggests that population‐specific genetic effects, likely mediated through environmental variation, contribute to life history development in rainbow trout.

## Graphical abstract

We find population‐specific genetic effects associated with life history development in two populations of rainbow trout. The figure shows a region of the genome—Chromosome 8—that seems to be especially important in migratory development in a population of trout from Alaska.

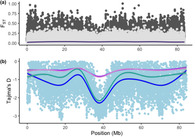

## 1 INTRODUCTION

The movement of animals to exploit seasonally available resources plays an important role in the development, maintenance, and overall health of many different ecosystems. This process, known as migration, is characterized by an onset of physiological and behavioral changes and is regulated by a combination of genetic factors and environmental cues (Åkesson & Hedenström, 2007; Liedvogel et al., 2011). Although migration occurs in many taxa, some species demonstrate a partially migratory life history, consisting of individuals who undergo the migratory process and those that remain as nonmigratory residents (Chapman et al., 2011). Although both behavioral types often coexist within the same ecosystem, the differences between the two ecotypes can lead to ecological and adaptive differences that are exclusive to each life history (Chapman et al., 2011; Gómez‐Bahamón et al., 2020; Winker, 2010). Anthropogenic disturbances, such as climate change, dam construction, and habitat modifications, are disproportionately affecting migratory individuals compared to resident conspecifics. Thus, providing an increased need to understand the extent to which migratory individuals are able to adapt to these changing environments and the underlying molecular mechanisms associated with these adaptations (Liedvogel et al., 2011; Singh & Milner‐Gulland, 2011; Wilcove & Wikelski, 2008).

Salmonids (salmon, trout, and charr) are an economically important group of fishes that are valued widely for both sportfishing and human consumption and play an important role in native subsistence fishing. One of the most abundant and well studied is the rainbow trout (*Oncorhynchus mykiss*), which is present in two distinct ecotypes; the rainbow (resident) and steelhead (migratory) trout. Heritability studies on *O*. *mykiss* have found strong evidence of an additive genetic component to migratory tendency (Hecht et al., 2015), and quantitative trait loci (QTL) studies have found loci on different linkage groups associated with multiple traits related to smoltification (the process by which resident individuals prepare for their anadromous migrations; Hecht et al., 2012; Le Bras et al., 2011; Nichols et al., 2008). More recently, genome‐wide association studies (GWAS) approaches have found associations between polymorphic positions within the rainbow trout genome and life history development (Arostegui et al., 2019; Hale et al., 2013; Hecht et al., 2013; Weinstein et al., 2019), suggesting that migratory behavior is a polygenic trait. Additionally, an inversion on chromosome 5 has been implicated in contributing to the migratory condition (Campbell et al., 2021; Pearse et al., 2019), especially in coastal populations from California and southern Oregon (but see Arostegui et al., 2019). However, many previous studies have used reduced‐representation sequencing methods (i.e., RAD‐seq and SNP chips) which leave large regions of the genome unqueried making it difficult to dissect the genetic architecture of this complex trait. Therefore, in order to accurately determine the genes and alleles associated with life history development, whole‐genome sequence data are required.

Systematically studying the genetic basis of migration in *O. mykiss* is challenged by accurately identifying migrants and residents within populations. This study capitalizes on information collected on residency and anadromy in two comprehensively studied systems: Sashin Creek and Little Sheep Creek. The Sashin Creek system, located in Southeastern Alaska, has become a model population to evaluate the genetic differentiation between anadromous and resident rainbow trout. This site contains a resident population formed by transplanting juveniles of mixed (anadromous and resident) parentage above two barrier waterfalls into Sashin Lake in 1926 (Thrower et al., 2004). These barriers allow juvenile smolts to leave the lake but prevent adult steelhead from returning to spawn, creating an allopatric resident population purged of most migratory alleles. Located below the barrier waterfalls in Sashin Creek is the ancestral population with access to the Pacific Ocean, which consists largely of migratory *O. mykiss*. Migratory and resident *O. mykiss* in this system have evolved broad genetic differences that appear to be associated with traits related to migration and smoltification (Hale et al., 2016; Hecht et al., 2015; McKinney et al., 2015; Thrower & Joyce, 2004; Weinstein et al., 2019). However, the previous studies in this system have used reduced‐representation genome methods and have not provided detailed genome‐wide data to assess the alleles and genes associated with migratory tendencies. Additionally, in‐depth analyses regarding the associations between migratory behavior, physiology, and genetics have been largely limited to the Sashin Creek system, making it impossible to test if, and to what extent, alleles associated with migration in Sashin Creek are shared with other populations (although see Campbell et al., 2021; Hecht et al., 2013; Pearse et al., 2019). To that end, we also investigated the genetic basis of anadromy in the Little Sheep Creek population in Oregon that lacks physical barriers to gene flow allowing both ecotypes to reproduce in the same environment (Berntson et al., 2011).

Herein, we use whole‐genome pooled‐sequencing (pooled‐seq) approaches to evaluate both the shared and population‐specific genetic components of migratory and resident life histories among two geographically separated populations of *O. mykiss* (Sashin Creek, AK and Little Sheep Creek, OR). These data were analyzed to locate (1) regions of the genome associated with anadromy, (2) compare the locations of these regions between the two populations studied, and (3) link these polymorphisms within underlying protein‐coding genes. The results contribute to an increasing body of information dedicated to understanding the shared and population‐specific genetic control of migratory behavior in *O*. *mykiss*.

## 2 MATERIALS AND METHODS

### 2.1 Sampling and DNA extraction

Resident rainbow and anadromous steelhead trout were sampled from two populations: Sashin Creek, Alaska and Little Sheep Creek (LSC), Oregon. In the Sashin system, all residents were sampled from Sashin Lake and all sampled migrants were adults returning to spawn in Sashin Creek. In the LSC system, migrants and residents coexist, so accurate phenotype determination was critical in classifying life history type. The migratory phenotype was determined using physical traits including silvery coloration and body shape, whereas residents were determined by the expression of gametes (as sexual maturity precludes anadromy in rainbow trout; more sampling details can be found in Berntson et al., 2011; Thrower et al., 2004). To prevent phenotyping of precocious juveniles, only adult fish with fork lengths (tip of snout to the fork of the caudal fin) greater than 150 mm were sampled. To ensure that only wild fish, and not hatchery fish, were collected for sampling, only fish with the full adipose fin were collected. At sampling, the fork length and sex of the fish were recorded. In the Sashin Creek system, samples were collected in the form of fin clips, and LSC samples included both fin clips and operculum punches; all samples were preserved in 95% ethanol. Fish were immediately released after processing.

### 2.2 Pooled‐sequencing library preparation

DNA was extracted from 174 fish—40 Sashin Creek migrants, 40 Sashin Creek residents, 48 LSC migrants, and 46 LSC residents using DNeasy Tissue Extraction kits (QIAGEN Corporation). Pooled‐seq was used to provide a cost and time‐efficient way of sequencing many individuals with a high depth of coverage. The utility of pooled‐seq methods in nonmodel organisms has been discussed in detail in Micheletti and Narum (2018). Pooled‐seq involves combining DNA from multiple samples of the same group into a singular pool which represents the population‐level allele frequencies (Schlötterer et al., 2014). To ensure equal representation of each individual in the pool, every DNA sample was normalized to a standard of 300 μg/L before combining samples into pools. Four DNA pools were prepared in total—Sashin migrants (SM), Sashin Residents (SR), Little Sheep Creek migrants (LSM), and Little Sheep Creek residents (LSR). Each pool contained an equal number of males and females. Pools were then sent to the NorthWest Genomics Center at the University of Washington (Sashin pools) and Novogene (LSC pools) for paired‐end 150 base pair sequencing on Illumina NovaSeq.

### 2.3 Pool‐Seq alignment and filtering

Sequences were analyzed following the *PoolParty* pipeline (https://github.com/StevenMicheletti/poolparty), as detailed in Micheletti and Narum (2018) with custom modifications. Sequences were first quality filtered to remove reads with poor quality bases (*Q* values <20) or that measured <50 base pairs in length. Quality‐filtered sequences were then aligned to the rainbow trout genome (Omyk_1.0; GCA_002163495.1) using *bwa mem* with default settings (Li & Durbin, 2009). Duplicate sequences were removed using *samblaster* (Faust & Hall, 2014), and *samtools* (Li et al., 2009; Li, 2011) was used to compile alignments and filter the reads to a minimum length of 36 and a quality score of >30 (Kofler et al., 2016). Alignments were then converted to the *mpileup* file format (in *samtools*), which summarizes total coverage information for each pool (Li et al., 2009) and then combined to a synchronized (sync) file to be used for analysis in *Popoolation2* (Kofler, Pandey, & Schloetterer, 2011) and *PoPoolations* (Kofler, Orozco‐terWengel, et al., 2011). Positions from each of the four pools were retained for use in statistical calculations if they fell between the minimum threshold of 25× depth of coverage, and the maximum threshold of 250× depth of coverage to reduce the chances of analyzing paralogs and sequences with low coverage. All candidate SNPs were required to have a minor allele frequency of at least 0.05 and each rare allele had to be present at least twice within each pool.

### 2.4 Bioinformatics and statistical calculations

Using the *Popoolation2* pipeline (Kofler, Pandey, & Schloetterer, 2011), Fixation index (*F*_ST_) values were calculated to evaluate genetic differentiation between resident and anadromous populations in both Sashin and LSC. Individual *F*_ST_ was calculated for each position within the genome using the *F*_ST_‐slide script in *Popoolation2* and the following parameters: ‐min‐count 8 ‐min‐coverage 25 ‐max‐coverage 250 ‐pool‐size 40. This value was calculated for each SNP individually to find SNPs with complete fixation (*F*_ST_ = 1) between migrant and resident pools. Manhattan plots were made using the program *CMplot* in R by plotting individual *F*_ST_ values at each position on the genome. Individual *F*_ST_ values were filtered to exclude any positions that produced *F*_ST_ values below 0.05 to reduce file size. Outlier loci were identified using the top 0.02% of *F*_ST_ values between migrant and resident pools in each population. To evaluate nucleotide diversity in each of the populations, a Tajima's *D* statistic was calculated over a 10,000 base pair window, with a step size of 10,000 for anadromous and resident Sashin and LSC pools using the variance program in *PoPoolations* (Kofler, Orozco‐terWengel, et al., 2011) using ‐min‐count 2 and ‐min‐qual 20. Using the same parameters as the Tajima's *D* statistic, Watterson's theta value was also calculated for each site using *Popoolations* to provide an estimate of the genetic diversity present between ecotypes from each location (Kofler, Orozco‐terWengel, et al., 2011). Theta estimates were determined also using a nonoverlapping window size of 10,000 base pairs.

## 3 RESULTS

**FIGURE 1:**
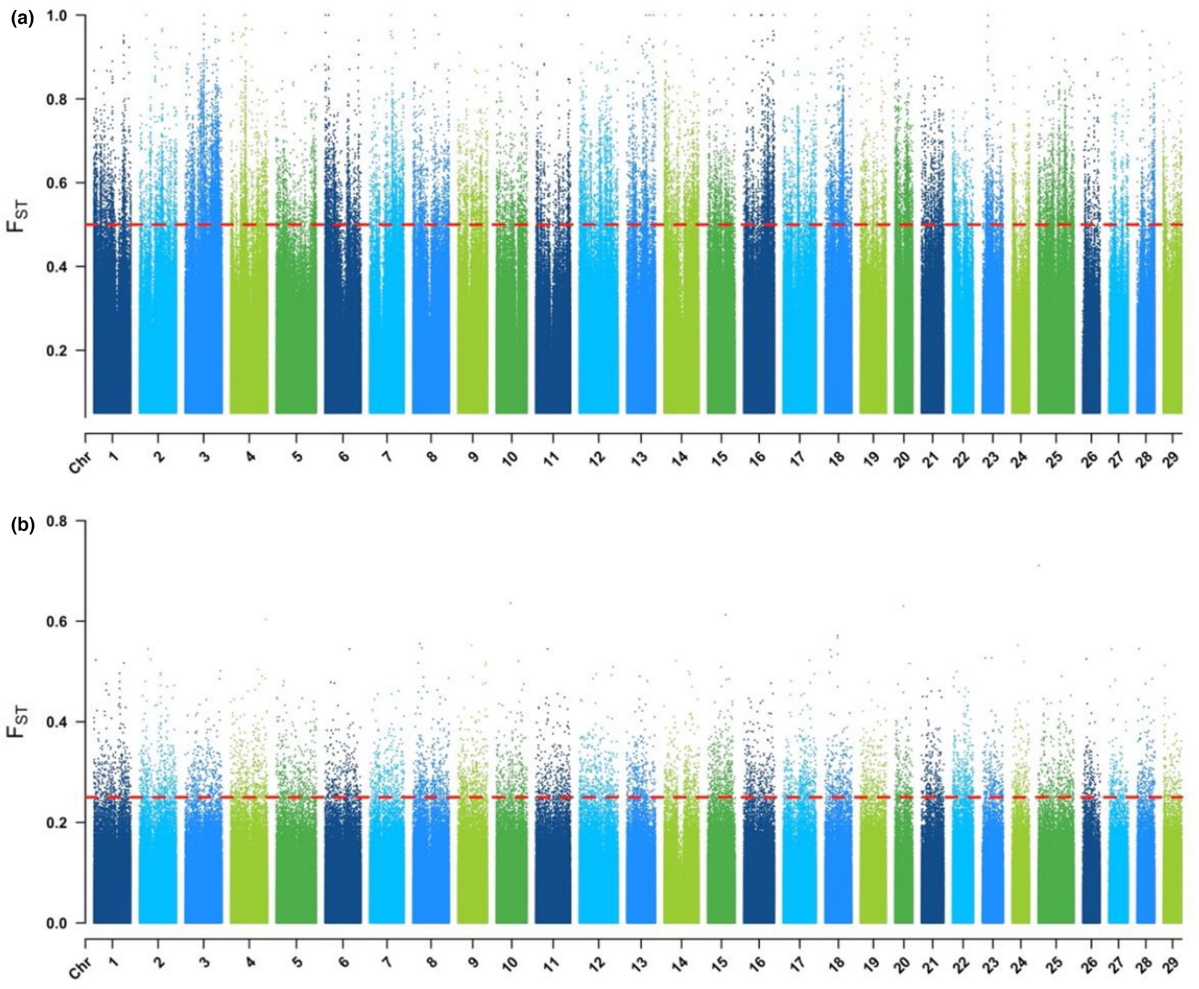
Individual fixation index (*F*_ST_) values for resident versus anadromous *Oncorhynchus mykiss* of (a) Sashin Creek and (b) Little Sheep Creek on a Manhattan plot. Each point on the plot represents an individual *F*_ST_ value, with a value of 1 indicating complete differentiation. The red line indicates the cut‐off value for the top 0.02% value for *F*_ST_ estimates.

Aligning the pooled sequencing to the rainbow trout genome produced an average depth per position of 30 reads for Little Sheep Creek and 115 reads for Sashin pools. A total number of quality‐filtered reads as well as average coverage depth are reported for each population—SM, SR, LSM, and LSR in Table 1. Filtering of candidate SNPs left a total of 16,432,862 SNPs between the Sashin Lake and Sashin Creek pools and 3,610,870 SNPs between the LSC resident and LSC migrant pools. SNP density varied throughout the genome with regions of high density being found at Chr1:81.6 Mb, Chr2:9–10 Mb, Chr14:22–25 Mb, Chr18:8–9 Mb, and Chr22: 44–46 Mb (see Figure S1). The genetic variance between ecotypes was analyzed by calculating the individual *F*_ST_ value for each SNP in Sashin and LSC populations (Figure 1). In the Sashin collection, 41 loci contained fixed differences between migrant and resident individuals (mean *F*_ST_ for all SNPs = 0.059 ± 0.076). There were no fixed differences identified in the LSC collection (mean *F*_ST_ = 0.029 ± 0.039). The top 1% *F*_ST_ quantile began at 0.355 and 0.186 and we used a cut‐off value of 0.02% to identify outlier *F*_ST_ values. This more conservative percentile produced 33,215 outlier loci (minimum *F*_ST_ of 0.5) in the Sashin population compared with 8667 loci (minimum *F*_ST_ 0.256) in LSC. Sashin Creek contained a greater density of polymorphic sites throughout the genome, likely due to both a higher depth of sequencing coverage and reduced gene flow between the two ecotypes. Regions of high *F*_ST_ density in both populations were identified at Chr1:81.6 Mb, Chr2:9–10 Mb, Chr14:22–25 Mb, Chr18:8–9 Mb, and positions Chr22:44–46 Mb (see Figure S1). There were 16 genome regions that showed a high‐individual *F*_ST_ in both Sashin and LSC that were further evaluated to detect if protein‐coding genes present in those regions may be the subject of selection (Table 2). Of these, only one SNP, located on chromosome 25, was within a protein‐coding region, found in RNA polymerase II‐associated protein one (*RPAP1*).

**TABLE 1:** Total number of quality‐filtered (QF) reads for each of the four populations, and the average depth of coverage of each pool assuming the published rainbow trout genome size of 2.1 Gb.

Location	Ecotype	Number of QF reads	Average depth of coverage
Sashin Lake	Rainbow	812,041,814	116.006
Sashin Creek	Steelhead	799,458,233	114.208
Little Sheep Creek	Rainbow	193,742,578	27.678
Little Sheep Creek	Steelhead	221,513,116	31.645

**TABLE 2:** Chromosome and base pair locations of shared SNPs showing elevated *F*_ST_ between anadromous and resident populations.

CHR	BP	LSC *F*_ST_	Sash *F*_ST_	Gene (if present)
1	13331	0.297	0.509	Phosphatidylinositol‐specific phospholipase C, X domain containing 1
1	69898903	0.291	0.560	—
3	74564628	0.316	0.684	—
4	34759942	0.275	0.930	Leptin receptor
5	80722208	0.278	0.520	DnaJ homolog, subfamily C, member 6
6	20756423	0.334	0.550	—
6	20755871	0.263	0.597	—
7	21949457	0.313	0.563	—
8	17055130	0.416	0.605	Tumor protein D53 homolog
13	37769240	0.255	0.601	cAMP‐specific 3′,5′‐cyclic phosphodiesterase 4B
19	19454334	0.256	1	Catenin alpha‐2
20	20645201	0.280	0.558	Transformation/transcription domain‐associated protein
22	47499934	0.268	0.519	—
25	51690773	0.335	0.503	RNA polymerase II‐associated protein 1
25	66224036	0.289	0.510	—
26	14291145	0.333	0.545	—

*Note*: *F*_ST_ values are listed for both Little Sheep Creek (LSC) and Sashin (Sash) populations. Know genes within 1 MB of the SNP are reported.

**FIGURE 2:**
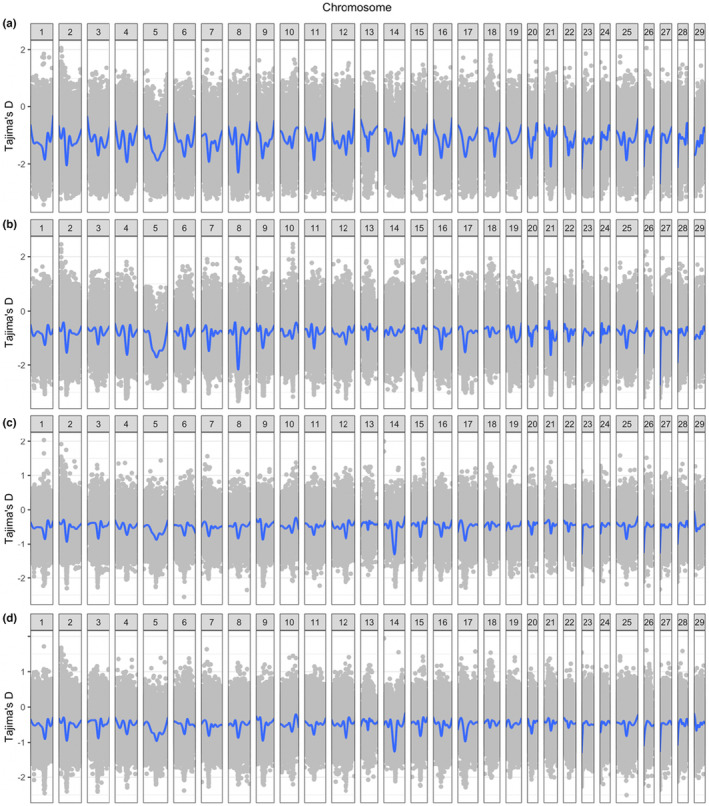
Tajima's *D* values for (a) Sashin residents, (b) Sashin migrants, (c) Little Sheep Creek residents, and (d) Little Sheep Creek migrants plotted in gray. Tajima's *D* was estimated over a nonoverlapping 10 kb window. Gray points represent individual estimates of Tajima's *D* within the 10 kb window. Blue lines represent the smoothed average Tajima's *D* values.

Tajima's *D* statistic was then calculated over adjacent 10,000 base pair windows across the genome of each of the four pools (Figure 2) to determine the type of selection event occurring in each window. We limited Tajima's *D* values to those that were greater than or equal to 2 or less than or equal to negative 2, which suggest a significant deviation from neutrality occurred (Tajima, 1989). This threshold identified 112 significant regions in the LSR collection, of which 94 positions were negative. In the SR collection, we identified 24,192 regions using the same parameters, of which all but three positions were negative. Between the two resident groups, 94 regions shared a significant value over the same window, and all were negative. In the migrant groups, we identified 222 significant Tajima's *D* values in the LSC migrant pool (no positive values), and 8853 in the SM pool (of which eight were positive). Of these, 151 were shared in the same window in both pools and were all negative. There were additionally 21 locations where the Tajima's *D* value was below negative 2 in all four pools, leaving 73 regions of low Tajima's *D* unique to the resident groups, and 130 regions of low Tajima's *D* unique to the migrants.

We also identified areas where there were significant Tajima's *D* values in adjacent shared windows (±10,000 base pairs), which may indicate a larger target area for selection. Any genes in these regions were noted for their corresponding protein function and related pathways. We additionally used these data to identify trends in both resident and migrant phenotypes where large regions of significant Tajima's *D* values were found close together, indicating a shared selection event acting on the phenotype. For the resident groups, these large regions of selection were located near Chr2:29–30 Mb, Chr3:38–39 Mb, Chr5:46–47 Mb, Chr9:26–27 Mb, Chr12:53–55 Mb, Chr14:43–44 Mb, Chr15:36–37 Mb, and Chr17:28–30 Mb. In the migrant pools, we identified regions near Chr1:50–52 Mb, Chr2:28–30 Mb, Chr5:47–48 Mb, Chr9:26–27 Mb, Chr15:35–37 Mb, Chr17:28–30 Mb, and Chr27: 0.3–1 Mb.

**FIGURE 3:**
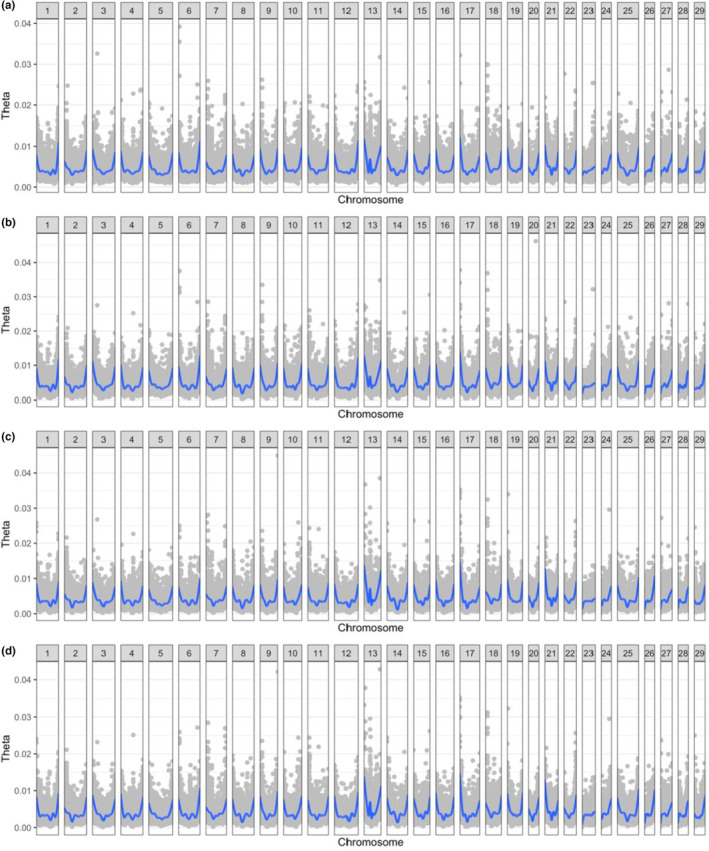
Watterson's theta values for (a) Sashin residents, (b) Sashin migrants, (c) Little Sheep Creek residents, and (d) Little Sheep Creek migrants plotted in gray. Watterson's theta was estimated over a nonoverlapping 10 kb window. Gray points represent individual estimates of Watterson's theta within the 10 kb window. Blue lines represent the smoothed average theta values.

The Watterson's theta estimate was calculated for each of the four populations, with a maximum theta value of 0.046 in the Sashin steelhead pool. Trends in diversity were compared between each of the populations to identify areas throughout the genome where increased polymorphic presence may contribute to a specific phenotype (Figure 3). We did not observe evidence for differences in nucleotide diversity based on either location or ecotype. Most peaks were observed near the telomeres, corresponding with increased recombination in those areas, but an area on chromosome 13 showed elevated theta near the centromere (Chr13:23–27 Mb). This peak corresponds with a location of increased *F*_ST_ density from both Sashin and Little Sheep Creek individuals, suggesting a possible area of importance for retaining genetic diversity within both populations.

## 4 DISCUSSION

The results presented herein indicate that the genetic architecture of life history development in *O. mykiss* is different in Sashin Creek and Little Sheep Creek. Although genetic control is important in life history development of migratory behavior in trout from Sashin Creek (and other populations; e.g., Arostegui et al., 2019; Campbell et al., 2021; Hecht et al., 2013; Pearse et al., 2019), the results from Little Sheep Creek suggest relatively little genetic differentiation between phenotypes. This discrepancy likely stems from topographic differences between the two systems as, unlike in the Sashin system, there are no physical barriers to gene flow between ecotypes in LSC. Unsurprisingly, this, combined with a major population bottleneck that occurred when the Sashin Lake system was founded, has led to a higher degree of genetic differentiation between ecotypes in Sashin compared with LSC. Although our findings suggest that the high degree of differentiation between life histories in Sashin Creek is due to genome‐wide genetic effects, there are notable differences between the results presented herein and previous studies regarding the regions of the genome associated with life history development. For example, regions on chromosomes 2, 7, 17, and 26 contained loci with high *F*_ST_ values between ecotypes in Sashin Creek (Hale et al., 2013; Weinstein et al., 2019), whereas our data suggested that these regions do not contain strongly differentiated loci. This discrepancy is most likely due to different biological samples being used as it is possible that there is a temporal aspect between alleles and life history development. It is known that environmental factors, such as temperature and water flow, have a strong effect on life history development in *O. mykiss* (Narum et al., 2008). Therefore, phenotypic plasticity may be a strong evolutionary force in determining life history development in rainbow trout, as many environmental cues are required for successful migratory events. Over time, this could lead to different alleles being associated with life history development in different years.

We identified 42 genomic positions with *F*_ST_ values of one between phenotypes in Sashin Creek, of which 20 were located within protein‐coding gene regions. Four were found within a 6000 base pair noncoding region of *leptin receptor* (*lepr*) on chromosome 4. There could be a connection between SNPs within *lepr* and migration as leptin modulates appetite and energy use (Gong et al., 2013; Murashita et al., 2008). Alterations in leptin receptor function or binding affinity between the receptor and leptin could be advantageous during periods with low food availability, and recent research indicates that leptin plays a role in migratory success (Choi et al., 2014; Fuentes et al., 2012). Leptin manages energy metabolism and body weight, both of which have been shown to be associated with migratory behavior through QTL studies (Hecht et al., 2012; Nichols et al., 2008). Moreover, samples from LSC also showed elevated *F*_ST_ values in SNPs located within *lepr* (chromosome 4; bp 34759942) suggesting this region of the genome may be under selection in *O. mykiss* more broadly. Although no SNPs were found in exon regions, these SNPs could be connected to differences in gene expression in *lepr*. Although previous RNA‐seq experiments using samples from Sashin Creek did not find *lepr* to be differentially expressed (Hale et al., 2016; McKinney et al., 2015), these studies sampled juveniles and so patterns of gene expression of *lepr* in adult trout have, to the best of our knowledge, not been measured.

Although most evidence points toward population‐specific genetic effects, we did uncover 16 SNPs with elevated differentiation in both Sashin and LSC. Eight SNPs were located within protein‐coding genes, but only a single SNP was found within a protein‐coding exon region, on *pap1 RNA polymerase II‐associated protein 1* (chr25; bp 51690773). However, this polymorphism was both synonymous and reversed in each pool, with the resident genotype in Sashin corresponding to the migrant genotype in LSC. Of the remaining 16 SNPs, six were found to have the same genotype distribution in both pools (i.e., the same alleles were associated with the same phenotype in both populations; Chr1:1331, Chr3: 74564628, Chr4: 34759942, Chr5: 80722208, Chr8: 17055130, Chr25: 66224036; see Table 2), and four of these were located within known genes. These genes are involved in a wide range of functions, including metabolism and protein synthesis, which could have downstream effects on smoltification or regulation of life history development (McCormick, 2013; Stefansson et al., 2012). Specifically, the gene *PI‐PLC* (*phosphatidylinositol‐specific phospholipase C, X domain containing 1* (chr1; bp 13331)) was found to segregate based on phenotypes in both LSC and Sashin. This gene encodes for phospholipase C involved in hydrolyzing phospholipids into fatty acids and other lipophilic molecules (reviewed by Fukami, 2002). Evidence for the connection between metabolism and migration is vast, and it has been suggested that standard metabolic rate may be a required threshold for smoltification in *O. mykiss*, as it is associated with dominance, food acquisition, and energy usage (Sloat & Reeves, 2014). Smolts alter their metabolic state in preparation for movement into seawater (McCormick, 2013; Stefansson et al., 2012), which is generally accompanied by a decrease in fatty stores, causing a change in condition factor that occurs in migratory salmonids before they leave freshwater (McCormick & Saunders, 1987; Sheridan, 1989). Therefore, polymorphisms within or nearby genes involved in metabolism might be under differential selection between *O. mykiss* ecotypes.

### 4.1 Nucleotide diversity in Sashin and Little Sheep Creek

**FIGURE 4:**
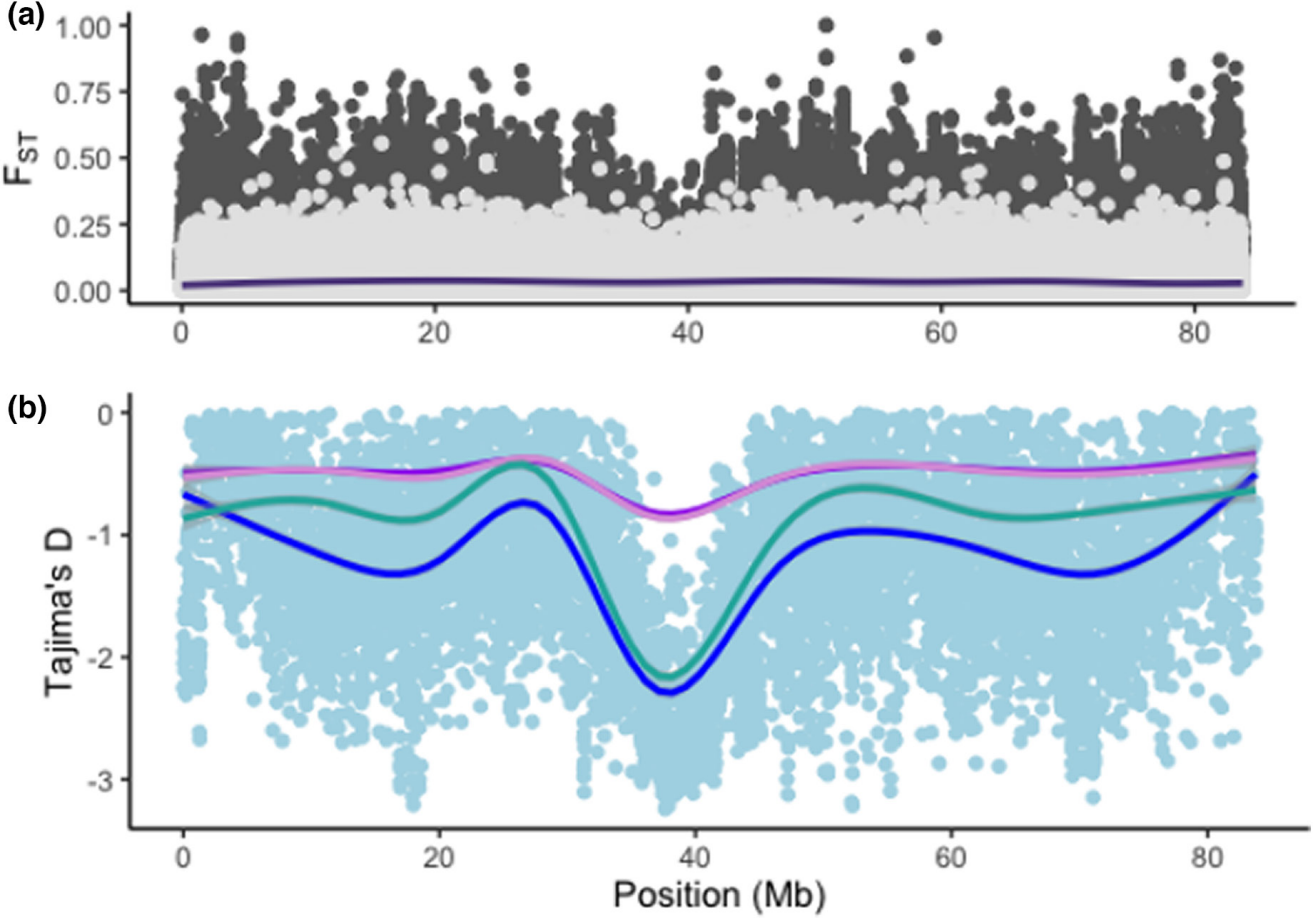
Estimates of *F*_ST_ and Tajima's *D* for chromosome 8. Panel a shows *F*_ST_ between ecotypes for both LSC (pale gray) and Sashin Creek (dark gray). The purple line shows the smoothed *F*_ST_ value for this region for the Sashin population. Panel b shows a marked reduction in *D* values between ~35 and 34 MB. Gray points represent individual estimates of Tajima's *D* from nonoverlapping 10 kb windows. Each line represents the smoothed Tajima's *D* values for rainbow trout (Sashin = pale blue, Little Sheep Creek = pale purple) and migratory steelhead trout (Sashin = dark blue, Little Sheep Creek = dark purple). Note that both Little Sheep Creek populations show very similar smoothed Tajima's *D* values, whereas both Sashin populations show a decline in estimates of *D*. Also, note that this reduction in *D* values is not associated with an increase in *F*_ST_ suggesting the alleles are shared between ecotypes in Sashin.

Overall measurements of nucleotide diversity show strong departures from neutrality for the Sashin Lake residents which may be indicative of underlying purifying selection (Thrower & Joyce, 2004). These patterns were not mirrored in LSC which showed Tajima's *D* values closer to neutrality (i.e., near 0). In populations, where ecotypes overlap in space and time, such as LSC, phenotypic plasticity may play a larger role in life history development than in populations where ecotypes are separated (Doctor et al., 2014; Dodson et al., 2013). Investigating Tajima's *D* values suggested that a ~10 Mb region on chromosome 8 (35–45 Mb) demonstrates purifying selection in both migrants and residents from Sashin Creek (Figure 4). Prior studies at Sashin have not suggested that this region of the genome is strongly associated with anadromy, but QTL studies in other populations have identified smoltification‐related traits that are localized to chromosome 8, including body morphology and increased growth and weight during the spring smoltification period (Nichols et al., 2008). This region also contained at least one gene with a known connection to photoperiod (s‐antigen visual arrestin; *sag*) and genes involved in photoperiod have been shown to be differentially expressed between life histories in Sashin Creek (Hale et al., 2016; McKinney et al., 2015). The Sashin Creek system is higher in latitude than LSC and therefore sensitivity to photoperiod—and a need for rapid growth in the short Alaskan summer—may explain why selection operating in this region of the genome is stronger in Sashin than in LSC. Although it is currently unknown if genes connected to photoperiod are also differentially expressed in other populations of *O. mykiss*.

A total of 10 genome regions were discovered that showed Tajima's *D* values less than −2 in both resident pools (Table 3). Although none of the genes located within these regions had previously been found to be associated with life history development in *O. mykiss*, proteins produced by *fibroblast growth factor 12* (*fgf12*) and *histone deacetylase 7* (*hdac7*) are connected to two genes (*fgfrl1* and *hdac11*) previously shown by Hale et al. (2016) to be differentially expressed between smolts and juvenile residents. These parallels could be indicative of the importance of cell signaling roles of fibroblast growth factors and potential protection from transcription factors by histone deacetylases. This could implicate epigenetic regulation or gene‐by‐environment interactions as significant contributing factors in the determination of life history type (Baerwald et al., 2016).

**TABLE 3:** Genome locations that produced Tajima's *D* values less than –2 in both resident populations.

CHR	MB	Gene	Protein function	Tajima's *D* (Sashin)	Tajima's *D* (LSC)
3	38.86–38.88	ptprub	Regulation of cell growth	−2.540 to −2.549	−2.019 to −2.119
3	39–39.02	—		−2.620 to −2.749	−2.002 to −2.022
3	39.12–39.15	—		−2.455 to −2.458	−2.045 to −2.141
3	39.39–39.41	hivep3	Transcription factor	−2.338 to −2.766	−2.050 to −2.080
5	47.91–47.94	acap2	Endocytosis and cell signaling	−2.479 to −2.740	−2.080 to −2.122
12	53.82–53.84	ssc4d	Scavenger receptor activity	−2.063 to −3.046	−2.086 to −2.117
15	36.06–36.09	mb21d2 fgf12	Cadherin binding Cell survival	−2.701 to −3.037	−2.003 to −2.051
17	29.79–29.82	hdac7	Histone activation	−2.034 to −2.450	−2.054 to −2.094

*Note*: Tajima's *D* values were calculated over a 10 kb window and details of protein‐coding genes within these windows are provided.

Regions of the genome suggesting convergent selection in migrants were identified by locating Tajima's *D* values greater than two or less than minus two in Sashin and LSC migrant pools (SM and LSM) that were not mirrored in residents (Table 4). As with the resident pools, all identified regions contained a negative Tajima's *D* which might be indicative of purifying selection (Tajima, 1989). A total of nine genes were identified over eight 10 kb regions with roles varying from protein interactions and binding to ion‐exchange capacity, again, supporting the conclusion that genes involved in life history development are many and varied. For example, the standard metabolic rate is often different between anadromous and resident salmonids (Sloat & Reeves, 2014), so the role of *ethanolamine‐phosphate phospho‐lyase* (*etnppl*) as a metabolic agent could be related to these adaptations and subsequent conversion of metabolic products. Several genes connected to ion binding and exchange were also found in regions of the genome with negative Tajima's *D* values (e.g., *eps1511*). This gene is especially interesting as ion exchange is crucial during smoltification (McCormick, 2013; Stefansson et al., 2012). To survive in freshwater, resident individuals are required to actively uptake ions (Na^2+^, Cl^−^, Ca^2+^) to compensate for passive ion loss (McCormick, 2013; Stefansson et al., 2012). However, in saltwater, this mechanism is reversed, and individuals are required to actively dispel those same ions to remain in homeostasis. Therefore, any mechanism that improves the function or transcription of genes involved in this process could be advantageous depending on the specific SNP change and the life history type of the individual.

**TABLE 4:** Genome locations that produced Tajima's *D* values less than −2 in both migratory populations.

CHR	MB	Gene	Protein function	Tajima's *D* (Sashin)	Tajima's *D* (LSC)
2	30.10–30.13	ankrd26	Protein–protein interactions	−2.370 to −2.882	−2.035 to −2.063
2	30.70–30.75	brsk2	Polarization of neurons and axonogenesis	−2.435 to −2.897	−2.007 to −2.044
5	47.36–47.39	agrd 2	Receptor and transmembrane signaling	−2.261 to −2.676	−2.027 to −2.035
9	26.11–26.17	etnppl col25a1	Metabolism and biosynthesis Extracellular matrix degradation	−2.339 to −2.620	−2.078 to −2.159
9	29.36–29.38	wnk2	Molecule transfer and transferase	−2.633 to −3.016	−2.077 to −2.101
23	0.43–0.46	sdf1	Receptor binding and chemokine activity	−2.047 to −2.874	−2.002 to −2.053
27	0.47–0.50	—		−2.471 to −2.586	−2.229 to −2.261
28	0.42–0.45	klf2 eps1511	Transcription factor Calcium ion bonding	−2.367 to −2.906	−2.001 to −2.119

*Note*: Tajima's *D* values were calculated over a 10 kb window and details of protein‐coding genes within these windows are provided.

Although there are many benefits to utilizing whole‐genome sequencing of *O*. *mykiss* to study the genetic basis of migration, limitations with pooled‐seq restrict our ability to determine the genotypes of the sequenced individuals. Nonetheless, the data generated herein add to our understanding of the genes and regions of the genomes important in life history development in rainbow trout. Perhaps, the most well‐documented genetic association with life history development in rainbow trout involves the chromosomal inversion located on chromosome 5 (Campbell et al., 2021; Pearse et al., 2014, 2019). The inversion follows a latitudinal cline and appears to be more important in life history development of southern, coastal locales than in the north. Moreover, the lack of association in this region of the genome with the data generated herein is unsurprising as previous studies failed to find the ancestral (or anadromous) form of the inversion in both Sashin and LSC (Hale et al., 2013; Weinstein et al., 2019). Taken together, studies strongly suggest that life history control in rainbow trout appears to be determined by population‐specific genetic effects (e.g., Hale et al., 2013; Hecht et al., 2013; Nichols et al., 2008) and the importance of different variants changes depending on the population in question.

Another consideration when utilizing the pooled‐seq methodology is the number of samples to combine per pool. These, together with the depth of sequencing coverage, are important because undersampling—either due to pooling too few individuals and/or due to low depth of coverage—can cause rare alleles to be missed. However, previous studies have simulated the number of samples required for accurate coverage of genetic variance. For example, Rellstab et al. (2013) report that between 10 and 20 individuals are sufficient to accurately estimate allele frequencies in natural populations. Similarly, Micheletti and Narum (whose PoolParty pipeline we used for the analyses of the data generated herein) suggest low‐coverage sequence data (i.e., 15× coverage) is sufficient to find the most rare alleles. Although more samples would increase the number of alleles discovered, such alleles will be rare and unlikely to contribute to the development of variable phenotypic traits (i.e., the overarching question of the research presented herein). Therefore, sampling at least 40 fish from each population and from each life history ecotype should have allowed for the accurate characterization of alleles within each population.

## 5 CONCLUSION

Our results support the idea that many alleles of small effect throughout the genome contribute to the development of migratory behavior in *O. mykiss* and that these alleles may be important in regulating gene expression. In addition, by comparing our results and the findings of prior studies with a collection from Little Sheep Creek, Oregon, we strongly suggest that the genetic basis of migration is controlled in a population‐specific manner, as only limited sharing of alleles exists between Sashin and LSC. However, we did identify eight shared regions of the genome which suggested purifying selection in both resident pools and eight other regions responding to selection in both pools of migratory steelhead trout. Although there is still much that is not understood about the biological regulation of migratory behavior, particularly in nonmodel species, our work contributes to a body of information uncovering the genetic control of the migratory process. To effectively evaluate this process, future research efforts should focus their efforts beyond the site in Sashin Creek, Alaska, as population‐specific effects likely play a role in regulating migratory behavior.

## AUTHOR CONTRIBUTIONS

**Catherine I. Clare:** Formal analysis (lead); methodology (lead); visualization (lead); writing – original draft (lead); writing – review and editing (lead). **Krista M. Nichols:** Conceptualization (supporting); methodology (supporting); project administration (supporting); writing – review and editing (supporting). **Frank P. Thrower:** Methodology (supporting); writing – review and editing (supporting). **Ewann A. Berntson:** Methodology (supporting); writing – review and editing (supporting). **Matthew C. Hale:** Conceptualization (lead); funding acquisition (lead); methodology (supporting); project administration (lead); supervision (supporting); writing – original draft (supporting); writing – review and editing (supporting).

## FUNDING INFORMATION

Funding for this research was provided by two TCU grants awarded to Matthew Hale.

## CONFLICT OF INTEREST STATEMENT

The authors declare that they do not have any conflicts of interest.

## Supporting information


Figure S1.

## Data Availability

Sequence data generated for this study have been deposited in the National Center for Biotechnology Information Sequence Read Archive under BioProject PRJNA978528.
