# Supplementary material for: Comparative genomics of rainbow trout (Oncorhynchus mykiss): Is the genetic architecture of migratory behavior conserved among populations?
Source: Ecol Evol. 2023 Jun 26;13(6):e10241. doi: 10.1002/ece3.10241 (PMC10293719; doi:10.1002/ece3.10241)
Supplement: Supplementary file 1 — Figure S1. [file ECE3-13-e10241-s001.pdf]

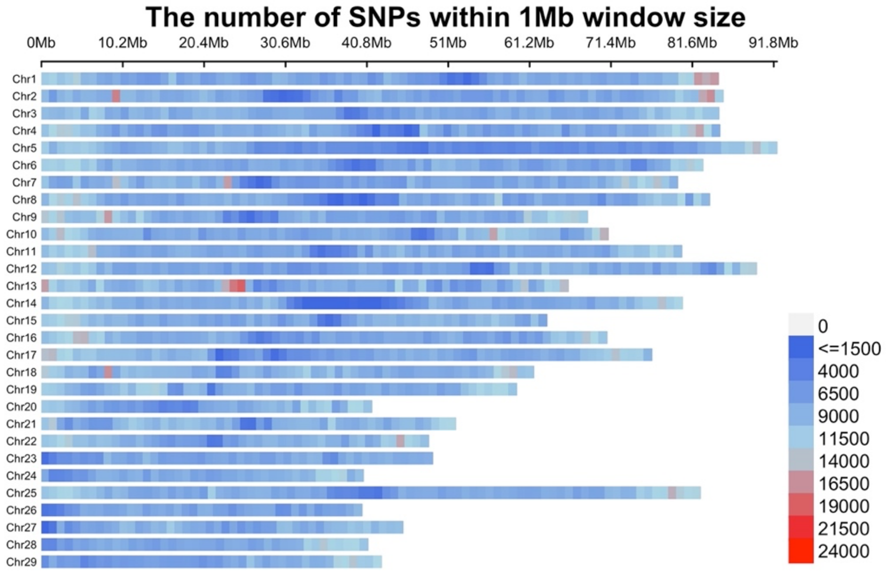

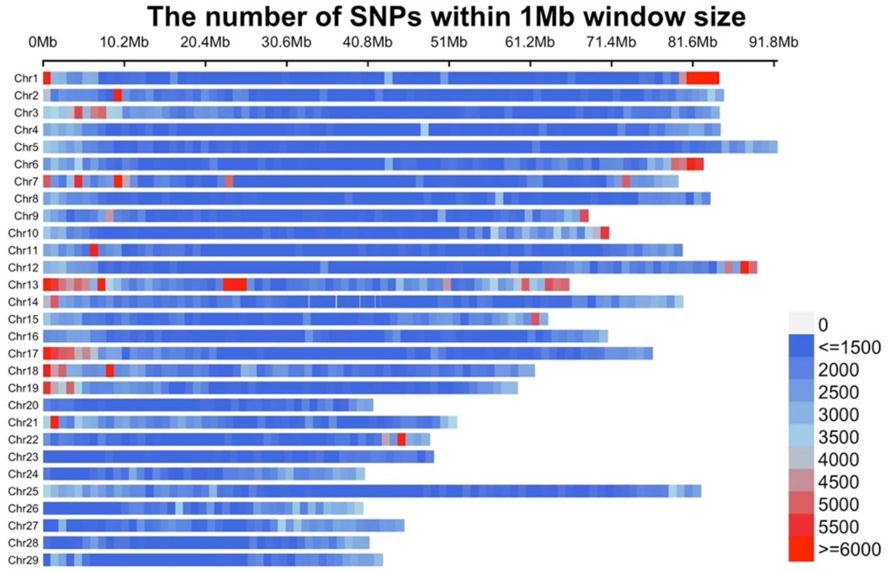

Supplemental Figure 1: SNPdensity for both Sashin (A) and Little Sheep Creek (B). Value is calculated from the number of SNPs over 1,000,000 base pair windows. Areas of low SNP density (Blue) indicate regions with either low sequencing coverage or substantial sequence overlap between migratory and resident O. mykiss, whereas red areas indicate regions of the genome with considerable differences between phenotypes.
